# Exploring *Urtica dioica* L. as a Promising Alternative Therapy for Obesity-Related Breast Cancer: Insights from Molecular Mechanisms and Bioinformatic Analysis

**DOI:** 10.1007/s11130-025-01341-8

**Published:** 2025-03-28

**Authors:** Ayla Eren, Mehmet Varol, Resat Unal, Filiz Altan

**Affiliations:** 1 Department of Molecular Biology and Genetics, Faculty of Science, Muğla Sıtkı Koçman University, 48000 Kötekli Muğla, Turkey; 2 Molecular Biology-Genetics and Biotechnology Program, Istanbul Technical University, 34469 Maslak Istanbul, Turkey

**Keywords:** *Urtica dioica* L., Breast cancer, Obesity, Molecular docking, Inflammation

## Abstract

Obesity and obesity-related breast cancer are major health problems that require alternative treatment strategies. *Urtica dioica* L. (*U. dioica*) stands out as a potential therapeutic candidate with its anti-oxidant, anti-cancer and lipid-lowering properties. In this study, the molecular effects of *U. dioica* were investigated by gene expression analysis and molecular docking methods. *U. dioica* significantly suppressed the expression of Brca1, Brca2, Fas, Lpl, Dgat1 and Mcp1 genes, resulting in significant changes in lipid metabolism, cancer susceptibility and inflammation. Molecular docking analyses showed that *U. dioica* components have strong binding affinities with target proteins. In particular, the interactions between Dgat1-Isorhamnetin rutinoside (-10.3 kcal/mol), Fas-Quercetin acetyl rutinoside (-10.3 kcal/mol), Lpl-Apigenin hexoside (-9.2 kcal/mol) and Mcp1-Quercetin acetyl rutinoside (-8.6 kcal/mol) were notable. In vitro and in silico analyses supported each other, revealing the effects of *U. dioica* in gene expression regulation and the potential for its constituents to interact with proteins. These findings indicate that *U. dioica* may be a promising alternative therapeutic agent in the treatment of obesity and obesity-related breast cancer and emphasize that its efficacy should be confirmed by clinical trials.

**Supplementary Information:**

The online version contains supplementary material available at 10.1007/s11130-025-01341-8.

## Introduction

Globally, the rate of obesity is on the rise and has reached pandemic levels, negatively affecting people’s health and quality of life [1]. The National Health Institutes and the World Health Organisation define obesity as having a body mass index (BMI) of more over 30 kg/m^2^ (30.0–34.9 is considered grade I obesity; 35.0–39.9 is considered grade II obesity; and 40 is considered grade III obesity). A difference between the amount of energy consumed through diet and the amount of energy expended through physical and metabolic activity typically causes a rise in BMI [2]. Breast cancer, the most common form of cancer in women, is significantly influenced by obesity. In this context, a protein known as breast cancer Type 1 susceptibility protein is encoded by the Brca1 gene, a human tumor suppressor gene [3]. Breast and ovarian cancer risk is higher in women who have mutant Brca1 or Brca2 genes. By the age of 70, female carriers of mutations in either Brca1 or Brca2 had a 60–80% likelihood of developing breast cancer [4]. Obesity exhibits a positive correlation with the expansion of adipose cells and an elevation in lipid accumulation. Among the enzymes crucial for triglyceride accumulation in fat tissue associated with obesity, Dgat1, Fas, and Lpl hold significant importance. The development of obesity in mice overexpressing Dgat1 in adipose tissue indicates the critical role Dgat1 plays in the development of obesity and insulin resistance [5]. Fas and Lpl provide non-esterified fatty acid substrates for triglyceride synthesis. Adipose tissue, the liver, and the lungs all have high levels of Fas expression, which controls the process of de novo lipogenesis from malonyl-CoA, acetyl-CoA, and NADPH [6]. Numerous human diseases and harmful health conditions, such as obesity, inflammation, cardiovascular disease, and most importantly cancer, have been connected to Fas in recent studies [7, 8]. The expression of Lpl is elevated in adipose and muscle tissues. Obesity raises the expression of both Lpl and Fas [9]. An important factor in the connection between obesity and breast cancer is inflammation. Chronic inflammation occurs in breast tissue due to fat accumulation caused by obesity [10] In this study, lipopolysaccharide (LPS) was used to trigger inflammation; high concentrations of LPS activated TLR4, ERK and NF-kB pathways, leading to IL-6 production and Mcp1 release [11]. A study that was published in the literature found that when adipocytes 3T3-L1 cells were stimulated with plant compounds, the treatment decreased the phosphorylation of ERK1/2, which is involved in the inflammation caused by LPS in adipocytes and increased the expression of pro-inflammatory genes associated with inflammation, including Mcp1, TNF-α, and IL-6. 3T3-L1 cells are the most widely characterized and frequently used cells in differentiation studies related to adipocytes [12].

Stinging nettle, also known as *U. dioica*, is a perennial medicinal plant species that is a member of the *Urticaceae* family. Recently, *U. dioica* has been researched for its anti-oxidant, anti-microbial, anti-ulcer, analgesic, and anti-cancer properties [13]. In addition, treatment with *U. dioica* extract was found to ameliorate insulin resistance induced by obesity [14]. In our investigation, we conducted an analysis of alterations in certain genetic markers modulated by molecular mechanisms connected with obesity and breast cancer, which we hypothesized to be associated with obesity, along with markers that promote lipid metabolism and heightened inflammation. The aim was to examine the potential therapeutic role of *U. dioica* at both the transcriptional and translational levels. To this end, we assessed the expression of genes such as Lpl, Fas, Dgat1, Brca1, Brca2, and Mcp1 in differentiated and undifferentiated cells, as well as in 3T3-L1 cells, treated with different concentrations and durations of *U. dioica* extract, utilizing quantitative polymerase chain reaction (qPCR). Subsequently, employing bioinformatics analysis via molecular docking, we obtained the most promising protein–ligand interaction revealing activation to observe if the plant extract has an effect on the above-mentioned genes.

## Materials and Methods

This section and also all the tables and figures are presented in the Supplementary Material.

## Results and Discussion

### Exploring the Influence of U. Dioica Extract on 3T3-L1 Adipocyte Viability

This study was designed to comprehensively evaluate the effects of *U. dioica* extracts on 3T3-L1 preadipocyte cells with different concentrations and durations. Cells were examined at doses ranging from 10–240 μg/ml and a concentration of 240 μg/ml was found to be toxic. In particular, concentrations of 50, 100 and 200 μg/ml were found to be consistent with the common dose ranges in the literature where the biological activities of *U. dioica* extracts have been examined. The 72-h period chosen in the study was determined to ensure a full understanding of potential cell damage and long-term effects, and the time periods of 24, 48 and 72 h used overlap with the periods commonly used in the literature [15]. Within the experimental framework involving 3T3-L1 preadipocytes, a systematic evaluation has been conducted to discern the impact of *U. dioica* extracts, dissolved in ethanol, at varying concentrations (50, 100, and 200 μg/ml) and durations (24, 48, and 72 h) on cell viability (Fig. S1). Intriguingly, a consistent attenuation of cell viability was evident at the 72-h mark, regardless of the administered concentration (Fig. S1c, S1f, S1i, S1l). Furthermore, a noteworthy observation was the manifestation of cellular detachment and morphological transformations at the highest concentration (200 μg/ml) after 48 and 72 h. Contrastingly, meticulous scrutiny revealed an absence of discernible alterations in cell surface adhesion at concentrations of 50, 100, and 200 μg/ml during the initial 24 h, and at 50 and 100 μg/ml during the subsequent 48 h, with no accompanying changes in cellular morphology. The administration of 50 and 100 μg/ml concentrations over 24 and 48 h emerged as the optimal conditions for preserving cell viability, as evidenced across all assessments (Fig. S1d, S1g, S1e, S1h). In the context of this in vitro study, 3T3-L1 preadipocyte cells served as a surrogate model and underwent stimulation with biologically relevant agents, including insulin, dexamethasone, and IBMX, to orchestrate their full differentiation into adipocytes. Over the orchestrated 12-day period, lipid droplet production was quantified utilizing the precise Oil Red O staining technique (see Fig. S2). This rigorous examination provides nuanced insights into the intricate interplay between *U. dioica* extracts and the nuanced cellular responses pertinent to adipogenesis. The findings are consistent with previous studies highlighting the biologically active properties of *U. dioica*. For example, *U. dioica* has been shown to be a potential source of natural therapy for the prevention or treatment of cancer, with anti-tumor, anti-metastatic, anti-proliferative and apoptotic effects on cancer cells [16]. The decrease in cell viability observed in this study is in line with previous reports, similar to high concentrations and prolonged administration. In a study it was revealed that *U. dioica* showed antiproliferative effects in breast cancer cell lines, triggered apoptosis and could inhibit tumor growth by 38% in in vivo tests by Karakol et al. [17]. In one study, *U. dioica* was shown to reduce Bcl-2, caspase-3 and caspase-9 activities in breast cancer and normal cells, affecting inflammation and cancer formation [18]. Furthermore, some studies have examined the relationship. Obanda et al. [14] reported that *U. dioica* extract decreased ceramide accumulation induced by FFA and by adiponectin mechanism. In addition, they reported that *U. dioica* inhibits high-fat diet-induced obesity and insulin resistance, which is related to its effects on lipid accumulation and glucose metabolism [19]. Regarding inflammation, *U. dioica* is known to have anti-inflammatory properties. Francišković et al. [20] found that *U. dioica* extracts show protective effects against inflammation and can treat conditions such as inflammatory bowel diseases.

### Effects of U. Dioica Extract on Gene Expression

The transcriptional analysis of the Fas, Lpl, and Dgat1 genes aimed to elucidate the impact of *U. dioica* extract on lipid and fat accumulation, as well as to comprehend the underlying molecular processes. Specifically, these genes (Dgat1, Lpl, and Fas) exhibit increased expression in the presence of the adipogenesis. Decreased Fas expression may not only reduce fatty acid synthesis but also limit the dependence of tumor cells on fatty acids as an energy source in breast cancer. Our study is in line with findings supporting the potential therapeutic use of Fas inhibitors [21]. Notably, these genes are known to upregulate in conditions mimicking obesity. After treating fully developed 3T3-L1 adipocyte cells for 24 h with 50 μg/ml extract, the level of Fas mRNA was reduced by 70% (Fig. S3a). While the mRNA level of Fas increased after 48 h of extract exposure, this elevation did not reach statistical significance. The enzyme Lpl plays a crucial role in converting triglycerides to fatty acids and glycerol, and its expression is heightened in obesity. Upon exposure to *U. dioica* extract at concentrations of 50 and 100 μg/mL for 48 h, a substantial reduction in Lpl mRNA expression was observed. Specifically, Lpl gene expression decreased by 80 and 90% at doses of 50 and 100 μg/ml, respectively (Fig. S3b). Similarly, decreases in Lpl and Dgat1 expressions play a critical role in the storage of triglycerides in adipose tissue and in energy metabolism. In relation to obesity, changes in Lpl activity may be an important factor in the development of fat accumulation and metabolic disorders. One study found that high Lpl activity was associated with adipocyte hypertrophy in subcutaneous adipose tissue, which reduced visceral fat accumulation and metabolic risk [22]. In another study, it was reported that Lpl activity in obese individuals increased after weight loss and that this increase played a role in the storage of triglycerides in adipose tissue [23]. The gene Dgat1, positively associated with obesity, exhibits heightened expression in obesity conditions. Treatment with *U. dioica* extract at concentrations of 50, 100, and 200 µg/ml for 24 h led to an 80, 80, and 90% reduction in Dgat1 gene expression, respectively (Fig. S3c). After 48 h, treatment at concentrations of 50 and 100 µg/ml resulted in a significant 80 and 85% decrease in Dgat1 gene expression, respectively. However, statistical analysis at a concentration of 200 µg/ml did not yield a significant result (Fig. S3d). In a study in the literature, it was reported that increased expression of Dgat1 in mice increased the triglyceride storage capacity of macrophages and provided protection against fatty acid-induced inflammatory activation [24]. In one study, Dgat1 targeting in glioblastoma cells was shown to inhibit lipid droplet formation, induce apoptosis and suppress tumor growth [25]. In our study, obesity inhibitory and anti-inflammatory effects of *U. dioica* were observed and results were consistent with these literature findings. In one study, diet supplemented with *U. dioica* was shown to reduce weight gain, fat accumulation in adipose tissue and insulin resistance. These findings support the positive effects of *U. dioica* on metabolic diseases, especially obesity and insulin resistance[19]. Breast cancer and obesity are known to be conditions in which inflammation enhances the development and accelerates the progression of the disease. LPS, found in the cell wall of gram-negative bacteria, shows strong proinflammatory effects. In a study, it was reported that *Alliin* (active component of *Allium sativum*) suppressed proinflammatory gene expression in LPS-stimulated adipocytes and decreased the expression of inflammation-related genes such as Mcp1, IL-6, Egr-1 [26]. The chemokine Mcp1 is known to affect macrophage accumulation and activity; in animal models, it is well recognised for its role as a marker of increased inflammation in adipose tissue linked to rising obesity. Notably, both obese humans and animals exhibit elevated expression levels of this gene in their adipose tissue. Following 24- and 48-h extract treatments, fully differentiated 3T3-L1 adipocyte cells displayed a noteworthy decrease in the expression of Mcp-1. Significantly reduced Mcp-1 mRNA expression was evident after cells underwent a 24-h treatment with *U. dioica* extract at concentrations of 50, 100, and 200 µg/ml. These results highlight a significant reduction in Mcp1 gene expression when fully differentiated adipocytes were treated with the extract for 24 h at doses of 50, 100, and 200 µg/ml (Fig. S3e). Comparable outcomes also showed a statistically significant decrease in Mcp1 gene expression following a 48-h treatment with extract at 50 and 100 µg/ml (Fig. S3f). In addition, Hoch et al. [27] reported that when obese individuals were administered 1 mg/mL LPS, inflammation-related IL-6, IL-2 and TNF-α cytokine levels increased in adipose tissue. *U. dioica* may reduce obesity-induced inflammation by suppressing the expression of inflammatory cytokines. In an in vitro study in the literature, *U. dioica* extract was found to suppress TNF-α and IL-1β release in human whole blood stimulated with LPS [28]. Our study confirms that *U. dioica* shows positive effects by inhibiting inflammatory pathways. Furthermore, *U. dioica* extract showed anti-inflammatory effects by suppressing NF-κB activation, reducing TNF-α and IL-1β production in synovial tissue [29]. Breast cancer development is more likely in women with early-stage diagnoses due in large part to genetic susceptibility, which is defined by mutations in the Brca1 and Brca2 genes. Hereditary gene mutations are thought to be responsible for 5–10% of instances of breast cancer, with the susceptibility genes Brca1 and Brca2 receiving particular attention. The increased risk of breast cancer associated with obesity has been studied in many studies, but the exact nature of this association is still unclear. In the study by Godet and Gilkes [30], mutations in the Brca1 and Brca2 genes were shown to predispose to breast cancer. Brca1’s mRNA expression pattern closely resembles Brca2’s, indicating that there is coordinated regulation at play here. Upon exposure to 50, 100, and 200 µg/ml extract for 24 h, the mRNA expression levels of both Brca1 and Brca2 genes exhibited a drastic reduction. However, after 48 h of extract treatment, a perceptible decrease in gene expression was observed, yet statistical analysis indicated that this reduction did not attain significance. These findings emphasize that the exposure of Brca1 and Brca2 genes, linked to breast cancer, to *U. dioica* extract concentrations of 50, 100, and 200 µg/ml for 24 h results in the anticipated effects. However, these effects are not sustained for 48 h, as statistical analysis indicates insignificance in the observed changes (Fig. S3g and S3h). (Table S3). *U. dioica* has been found to lead to reductions in transcriptional level in both 3T3-L1 adipocytes in obesity and in a breast cancer cell line in a breast cancer study [14, 18]. In vivo experiments on mice have shown that *U. dioica* has a protective effect against obesity when consumed as a vegetable [19]. Alternatively, they suggested that *U. dioica* inhibits cell proliferation and reduces cancer cell viability by inhibiting phosphorylation of PI3K/AKT pathway elements [31]. In addition, a study revealed that *U. dioica* selectively targets the JAK2/STAT3 pathway in breast cancer, showing strong binding affinity to JAK2 and anti-cancer potential [32].

## Molecular Docking Findings

With the developing technology, studies at the molecular level make it possible to examine the interactions, binding energies and biological activities of molecules through computer simulations [33]. This provides an important advance in the field of pharmacology, where stable structures of receptor-ligand interactions can be determined using Newtonian dynamics and thermodynamic calculations; low binding energies indicate high binding affinity and strong interaction. The molecular docking analysis conducted in this study provided key insights into the interaction dynamics of *U. dioica*-derived ligands with the dedicated crucial proteins implicated in obesity-related pathways such as metabolic, inflammatory, and oncological pathways. Binding affinities were evaluated, and the results underscored significant ligand–protein binding events, as summarized in Table S4. The most noteworthy docking results, illustrating optimal interactions, are outlined in Table S5 and Fig. S4. Dgat1 exhibited the highest binding affinity (−10.3 kcal/mol), followed closely by Fas (−10.3 kcal/mol) and Lpl (−9.2 kcal/mol). Furthermore, Quercetin acetyl rutinoside exhibited a binding energy of (−8.6 kcal/mol) when interacting with the Mcp-1 protein at this particular juncture. Comparatively lower binding energies were observed for Brca1 (−7.5 kcal/mol) and Brca2 (−8.4 kcal/mol) with apigenin hexoside. These variations in binding affinity can be attributed to differences in binding site topography and amino acid composition.

Hydrogen bonds, pi-π and pi-alkyl interactions stabilize the binding energy in protein–ligand interactions, allowing a better understanding of molecular interactions in a biological context. Özgen and Ünlü [34] emphasized the contribution of hydrogen bonds, pi-cation and pi-alkyl interactions in the binding stability of interactions by molecular docking method. In our study, Dgat1, a pivotal enzyme in triglyceride synthesis, demonstrated robust interactions with isorhamnetin rutinoside and quercetin acetyl rutinoside, evidenced by a binding energy of −10.3 kcal/mol. The key stabilizing forces were conventional hydrogen bonds with TRP C:377, ASN C:378, CYS C:385, and HIS C:415 (Table S4). Additionally, carbon-hydrogen bonding with GLN C:375 contributed to the binding stability. Noteworthy were the π–π stacking interactions with TRP C:374 and TRP C:377, which significantly enhance the binding free energy by providing favorable π-electron cloud overlap (Fig. S4c). These interactions not only stabilize the ligand within the hydrophobic core of Dgat1 but also suggest a mechanism by which these ligands may inhibit triglyceride biosynthesis. Given Dgat1’s role in adipogenesis, these findings support its therapeutic potential in obesity management. The interaction of quercetin acetyl rutinoside with Fas resulted in a high binding energy of −10.3 kcal/mol. The ligand formed multiple hydrogen bonds with key residues ARG A:2275, GLU A:2395, and SER A:2417 (Fig. S4b). The presence of π–π stacking with TYR B:2425 and π-alkyl interactions involving LEU A:2279 and PHE A:2418 provided additional stabilization. Importantly, the interaction profile highlighted the critical role of aromatic and hydrophobic residues in ligand binding. The π-electron cloud interactions likely enhance ligand specificity and retention, potentially leading to allosteric inhibition of Fas activity. As Fas overexpression is linked to various cancers, particularly breast cancer, these findings underscore the therapeutic relevance of *U. dioica*-derived ligands in oncological contexts. There is no direct reference to a specific molecular docking study examining the interactions of quercetin acetyl rutinoside with the Fas enzyme. However, in a study on quercetin and its derivatives, quercetin, which is among the active components of *Tamarindus indica*, was subjected to molecular docking analysis with three different domains of Fas. This study demonstrated that quercetin may have a potential inhibitory effect on the Fas enzyme [35]. Apigenin hexoside displayed a notable binding energy of −9.2 kcal/mol with Lpl, a key enzyme involved in lipid metabolism (Fig. S4f). Conventional hydrogen bonds with residues SER A:446, ARG A:447, and GLU A:448 were identified, alongside π-anion interactions with GLU A:448 and π-sigma interactions with VAL A:461. These electrostatic and hydrophobic interactions likely enhance the ligand’s binding affinity and specificity. The stabilization of Lpl by apigenin hexoside suggests a potential mechanism for enhancing lipid hydrolysis, thereby reducing circulating triglyceride levels. This interaction profile positions apigenin hexoside as a promising candidate for managing hyperlipidemia and related metabolic disorders.The interaction of quercetin acetyl rutinoside with Mcp1 yielded a binding energy of −8.6 kcal/mol (Table S4). Conventional hydrogen bonding with LYS B:38, along with carbon-hydrogen bonds involving LYS A:35 and PRO A:37, provided a stable interaction network (Fig. S4e). Additionally, a π–π stacking interaction with TYR A:13 and π-alkyl interactions with LYS B:35 and PRO B:37 were observed. The π-stacking interactions may play a pivotal role in ligand stabilization within the Mcp1 binding site. Given the role of Mcp-1 in monocyte recruitment and inflammation, these interactions suggest that quercetin acetyl rutinoside may serve as an anti-inflammatory agent by modulating Mcp-1 activity. This could have implications for conditions characterized by chronic inflammation. Apigenin hexoside exhibited moderate binding energies of −7.5 kcal/mol and −8.4 kcal/mol with Brca1 and Brca2, respectively (Table S4). In Brca1, hydrogen bonds were established with SER A:1655, LEU A:1657, and ASN A:1678, while π-cation and π-alkyl interactions involved LYS A:1702 and LEU A:1701 (Fig. S4a). Similarly, in Brca2, hydrogen bonds formed with HIS A:294, SER A:296, and THR A:297, complemented by π-donor hydrogen bonds with SER A:317 and π-alkyl interactions with ILE A:314 and ALA A:323 (Fig. S4d). Although the binding energies were lower than those for Dgat1, Fas, and Lpl, the interactions observed suggest potential modulation of Brca-mediated DNA repair pathways. This may have therapeutic implications in cancer, particularly in sensitizing cancer cells to DNA-damaging agents.

The strong binding affinities of isorhamnetin rutinoside and quercetin acetyl rutinoside with Dgat1 and Fas support their potential as inhibitors of triglyceride and fatty acid synthesis, respectively. Such inhibition may be particularly valuable in treating obesity and related metabolic disorders. The interaction of apigenin hexoside with Lpl suggests its role in enhancing lipid catabolism, potentially lowering plasma triglyceride levels and mitigating hyperlipidemia. Additionally, the modulation of Mcp1 activity by quercetin acetyl rutinoside implies an anti-inflammatory effect, which could be beneficial in managing chronic inflammatory conditions. Furthermore, the interactions of apigenin hexoside with Brca1 and Brca2 proteins indicate a potential role in modulating DNA repair pathways. This may enhance the efficacy of existing chemotherapeutic agents by increasing cancer cell sensitivity to DNA damage.

## Conclusions

Obesity has emerged as a complex health condition with inflammatory implications, exacerbating the risks of diseases such as breast cancer, Type-II diabetes, and cardiovascular disorders. Medicinal plants, such as *U. dioica*, have gained attention for their potential in mitigating obesity and breast cancer. Research findings suggest that *U. dioica* extract modulates key molecular pathways associated with obesity and breast cancer, leading to the downregulation of critical markers like Dgat1, Lpl, Fas, Mcp1, Brca1, and Brca2 in LPS induced adipocytes. Molecular docking studies further support the effectiveness of *U. dioica* components in interacting with specific proteins associated with breast cancer susceptibility and adipocyte differentiation. The integration of molecular docking results and quantitative PCR analyses provides a comprehensive understanding of *U. dioica*’s impact at both transcriptional and protein interaction levels. In conclusion, *U. dioica* presents itself as a valuable natural resource in the pursuit of effective and minimally invasive treatments for obesity and obesity-related breast cancer. Research on the molecular pathways that *U. dioica* influences will surely lead to the creation of new treatment approaches that might completely alter the way we tackle these widespread health issues. Future research should focus on validating the therapeutic potential of *U. dioica* with in vivo models. Clinical trials are critical to evaluate the safety and efficacy of *U. dioica* in humans. In addition to confirming the therapeutic potential of *U. dioica*, these studies will contribute to the development of targeted therapeutics for obesity and breast cancer. In addition to confirming the therapeutic potential of *U. dioica*, these studies will contribute to the development of targeted therapeutics for obesity and breast cancer.

## Supplementary Information

Below is the link to the electronic supplementary material.

Supplementary file1 (DOCX 2.24 MB)

## Data Availability

No datasets were generated or analysed during the current study.

## References

[1] Urhan M, Akbulut G, Ruh M et al (2017) Obesity and cancer relationship: Is leptin a carcinogenic adipokine? İzmir Katip Çelebi Univ Faculty Health Sci J 2:35–43

[2] Andò S, Gelsomino L, Panza S et al (2019) Obesity, Leptin and Breast Cancer: epidemiological evidence and proposed mechanisms. Cancers 11:62. 10.3390/CANCERS11010062 30634494 10.3390/cancers11010062 PMC6356310

[3] Miki Y, Swensen J, Shattuck-Eidens D et al (1994) A strong candidate for the breast and ovarian cancer susceptibility gene BRCA1. Science (1979) 266:66–71. 10.1126/SCIENCE.7545954 10.1126/science.7545954 7545954

[4] Smith RA, Cokkinides V, Brawley OW (2009) Cancer screening in the United States, 2009: a review of current American Cancer Society guidelines and issues in cancer screening. CA Cancer J Clin 59:27–41. 10.3322/CAAC.20008 19147867 10.3322/caac.20008

[5] Chen HC, Stone SJ, Zhou P et al (2002) Dissociation of obesity and impaired glucose disposal in mice overexpressing Acyl Coenzyme A: Diacylglycerol Acyltransferase 1 in white adipose tissue. Diabetes 51:3189–3195. 10.2337/DIABETES.51.11.3189 12401709 10.2337/diabetes.51.11.3189

[6] Doolittle MH, Ben-Zeev O, Elovson J et al (1990) The response of lipoprotein lipase to feeding and fasting. Evidence for posttranslational regulation. J Biol Chem 265:4570–4577. 10.1016/S0021-9258(19)39601-2 2307676

[7] Angeles TS, Hudkins RL (2016) Recent advances in targeting the fatty acid biosynthetic pathway using fatty acid synthase inhibitors. Expert Opin Drug Discov 11:1187–1199. 10.1080/17460441.2016.1245286 27701891 10.1080/17460441.2016.1245286

[8] Deepa PR, Vandhana S, Krishnakumar S (2013) Fatty acid synthase inhibition induces differential expression of genes involved in apoptosis and cell proliferation in ocular cancer cells. Nutr Cancer 65:311–316. 10.1080/01635581.2013.748923 23441619 10.1080/01635581.2013.748923

[9] Semenkovich CF (1997) Regulation of fatty acid synthase (FAS). Prog Lipid Res 36:43–53. 10.1016/S0163-7827(97)00003-9 9373620 10.1016/s0163-7827(97)00003-9

[10] Crespi E, Bottai G, Santarpia L (2016) Role of inflammation in obesity-related breast cancer. Curr Opin Pharmacol 31:114–122. 10.1016/J.COPH.2016.11.004 27889687 10.1016/j.coph.2016.11.004

[11] Park MY, Mun ST (2014) Carnosic acid inhibits TLR4-MyD88 signaling pathway in LPS-stimulated 3T3-L1 adipocytes. Nutr Res Pract 8:516. 10.4162/NRP.2014.8.5.516 25324930 10.4162/nrp.2014.8.5.516 PMC4198963

[12] Sarjeant K, Stephens JM (2012) Adipogenesis Cold Spring Harb Perspect Biol 4:a008417. 10.1101/CSHPERSPECT.A008417 22952395 10.1101/cshperspect.a008417 PMC3428766

[13] Fattahi S, Ardekani AM, Zabihi E et al (2013) Antioxidant and apoptotic effects of an aqueous extract of Urtica dioica on the MCF-7 human breast cancer cell line. Asian Pac J Cancer Prev 14:5317–5323. 10.7314/APJCP.2013.14.9.5317 24175819 10.7314/apjcp.2013.14.9.5317

[14] Obanda DN, Zhao P, Richard AJ et al (2016) Stinging Nettle (Urtica dioica L.) Attenuates FFA induced ceramide accumulation in 3T3-L1 adipocytes in an adiponectin dependent manner. PLoS One 11:e0150252. 10.1371/JOURNAL.PONE.0150252 26939068 10.1371/journal.pone.0150252 PMC4777364

[15] Kaya H, Tokgöz HB, Unal R, Altan F (2023) The effects of the Rheum ribes plant extract on inflammation, extracellular matrix remodeling, and obesity suggest a therapeutic potential. Mol Biol Rep 50:5223–5232. 10.1007/S11033-023-08478-2 37126207 10.1007/s11033-023-08478-2

[16] Esposito S, Bianco A, Russo R et al (2019) Therapeutic perspectives of molecules from urtica dioica extracts for cancer treatment. Molecules 24:2753. 10.3390/MOLECULES24152753 31362429 10.3390/molecules24152753 PMC6695697

[17] Karakol P, Saraydin S et al (2022) Anticancer effects of *Urtica dioica* in breast cancer. Asian Pacific journal of cancer prevention: APJCP, 2022 10.31557/APJCP.2022.23.2.673 PMC9272626 35225481

[18] Mohammadi A, Mansoori B, Baradaran PC et al (2017) *Urtica dioica* Extract Inhibits Proliferation and Induces Apoptosis and Related Gene Expression of Breast Cancer Cells *in Vitro* and *in Vivo*. Clin Breast Cancer 17:463–470. 10.1016/j.clbc.2017.04.008 28501502 10.1016/j.clbc.2017.04.008

[19] Fan S, Raychaudhuri S, Kraus O et al (2020) *Urtica dioica* whole vegetable as a functional food targeting fat accumulation and insulin resistance-a preliminary study in a mouse pre-diabetic model. Nutrients 12. 10.3390/nu12041059 10.3390/nu12041059 PMC7231388 32290353

[20] Francišković M, Gonzalez-Pérez R, Orčić D et al (2017) Chemical composition and immuno-modulatory effects of *Urtica dioica* L. (Stinging Nettle) extracts. Phytother Res 31:1183–1191. 10.1002/PTR.5836 28544187 10.1002/ptr.5836

[21] Ranganathan G, Unal R, Pokrovskaya I et al (2006) The lipogenic enzymes DGAT1, FAS, and LPL in adipose tissue: effects of obesity, insulin resistance, and TZD treatment. J Lipid Res 47:2444–2450. 10.1194/jlr.M600248-JLR200 16894240 10.1194/jlr.M600248-JLR200 PMC1850099

[22] Serra MC, Ryan AS, Sorkin JD et al (2015) High adipose LPL activity and adipocyte hypertrophy reduce visceral fat and metabolic risk in obese, older women. Obesity 2015(23):602–607. 10.1002/oby.20998 10.1002/oby.20998 PMC4340730 25612068

[23] Schwartz R, Brunzel JD (1981) Increase of adipose tissue lipoprotein lipase activity with weight loss. J Clin İnvestig 67(5):1425–1430 7229033 10.1172/JCI110171 PMC370709

[24] Koliwad SK, Streeper RS, Monetti M et al. (2010). DGAT1-dependent triacylglycerol storage by macrophages protects mice from diet-induced insulin resistance and inflammation. Am Soc Clin Investig 120. 10.1172/JCI36066 10.1172/JCI36066 PMC2827941 20124729

[25] Cheng X, Geng F, Pan M et al (2020) Targeting DGAT1 ameliorates glioblastoma by increasing fat catabolism and oxidative stress. Cell Metab 32(2):229–242 32559414 10.1016/j.cmet.2020.06.002 PMC7415721

[26] Quintero-Fabián S, Ortuño-Sahagún D, Vázquez-Carrera M, López-Roa RI (2013) Alliin, a garlic (Allium sativum) compound, prevents LPS-induced inflammation in 3T3-L1 adipocytes. Mediators Inflamm 2013. 10.1155/2013/381815 10.1155/2013/381815 PMC3888727 24453416

[27] Hoch M, Eberle AN, Peterli R et al (2008) LPS induces interleukin-6 and interleukin-8 but not tumor necrosis factor-alpha in human adipocytes. Cytokine 41:29–37. 10.1016/J.CYTO.2007.10.008 18060802 10.1016/j.cyto.2007.10.008

[28] Devkota HP, Paudel KR, Khanal S et al (2022) Stinging nettle (Urtica dioica L.): nutritional composition, bioactive compounds, and food functional properties. Molecules 27:5219. 10.3390/MOLECULES27165219 36014458 10.3390/molecules27165219 PMC9413031

[29] Taheri Y, Quispe C, Jes J et al (2022) Urtica dioica-derived phytochemicals for pharmacological and therapeutic applications. Evid Based Complement Altern Med 2022(16):20. 10.1155/2022/4024331 10.1155/2022/4024331 PMC8894011 35251206

[30] Godet I, M. Gilkes D (2017) BRCA1 and BRCA2 mutations and treatment strategies for breast cancer. Integr Cancer Sci Ther 4. 10.15761/icst.1000228 10.15761/ICST.1000228 PMC5505673 28706734

[31] Wenyuan JI, Shaoyin WEI, Wei LIU (2021) Effects of *Urtica dioica* extract on malignant biological behaviors of breast cancer cells and its possible mechanism. Chin J Cancer Biother 28(8).

[32] Upreti S, Muduli K, Pradhan J, et al (2023) Identification of novel inhibitors from Urtica spp against TNBC targeting JAK2 receptor for breast cancer therapy. Med Oncol 40. 10.1007/S12032-023-02193-5 10.1007/s12032-023-02193-5 37806999

[33] Hassan Baig M, Ahmad K, Roy S et al (2016) Computer aided drug design: success and limitations. Curr Pharm Des 22(5):572–581 26601966 10.2174/1381612822666151125000550

[34] Özgen A, Ünlü N (2022) Investigation of the interaction of a bacterial signal complex and peonidine molecule by molecular docking method. Fırat Univ J Sci 34:201–206

[35] Putri C, Sumitro S, Hayati SK-BP (2017) Quercetin, rutin, proanthocyanidin, catechin and epitacethin as fatty acid synthase inhibitor using virtual screening. 10.23869/bphjbr.23.1.20175

