# Supplementary material for: Exploring Urtica dioica L. as a Promising Alternative Therapy for Obesity-Related Breast Cancer: Insights from Molecular Mechanisms and Bioinformatic Analysis
Source: Plant Foods Hum Nutr. 2025 Mar 28;80(2):102. doi: 10.1007/s11130-025-01341-8 (PMC11953088; doi:10.1007/s11130-025-01341-8)
Supplement: Supplementary file 1 — Supplementary file1 (DOCX 2.24 MB) [file 11130_2025_1341_MOESM1_ESM.docx]

**Exploring *Urtica dioica* L. as a Promising Alternative Therapy for Obesity-Related Breast Cancer: Insights from Molecular Mechanisms and Bioinformatic Analysis**

**Ayla EREN^1,2^**

<https://orcid.org/0000-0002-5521-6402>

eren23@itu.edu.tr

**Mehmet VAROL^1^**

<https://orcid.org/0000-0003-2565-453X>

[mehmetvarol@mu.edu.tr](mailto:mehmetvarol@mu.edu.tr)

**Reşat ÜNAL^1^**

<https://orcid.org/0000-0002-1365-2310>

[resatunal@mu.edu.tr](mailto:resatunal@mu.edu.tr)

**Filiz ALTAN^1,^***

<https://orcid.org/0000-0001-6358-2448>

[afiliz@mu.edu.tr](mailto:afiliz@mu.edu.tr)

^1^ Department of Molecular Biology and Genetics, Faculty of Science, Muğla Sıtkı Koçman University, 48000, Kötekli, Muğla, Turkey

^2^ Istanbul Technical University, Molecular Biology-Genetics and Biotechnology Program, 34469, Maslak, Istanbul, Turkey

***Corresponding author:** [**afiliz@mu.edu.tr**](mailto:afiliz@mu.edu.tr)

**Supplementary Material**

**Materials and Methods**

**Preparation of above-ground parts of plant material**

*U. dioica*, a plant belonging to the *Urticaceae* family and characterized by stinging hairs, was harvested from the Muğla/Koycegiz region during both spring and autumn seasons when the specimens were in their optimal freshness. Following collection, the plants underwent a two-week drying process in a cool, dark environment. The aboveground part of the dried plant powders was ground and stored in pure ethanol solution for 7 days [1]. After 7 days, the plant extract was filtered and an evaporator was used to remove the solvent. The stock solution was prepared with a final concentration of 20 mg/ml in ethanol and stored at +4°C for use in the experiment.

**3T3-L1 preadipocyte cell cultivation and adipogenic differentiation**

3T3-L1 preadipocyte cells, derived from fibroblasts, were obtained from the American Type Culture Collection (ATCC) and cryopreserved in cryovials for subsequent use in cell culture experiments. Cells were inoculated into flasks to induce the differentiation of 3T3-L1 preadipocytes. After the cells adhered to the flask surface and reached growth inhibition, the old culture medium was removed. The preadipocytes were cultured in Dulbecco's Modified Eagle's Medium (DMEM; L0102-500) supplemented with 10% Neonatal Calf Serum (Capricorn, NCS-1B) and 1% Penicillin-Streptomycin (Multicell, 450-201-EL) at 37°C in a humidified atmosphere with 5% CO₂. The culture medium was replaced every other day, and the cell density was monitored microscopically. For adipogenic differentiation, DMEM supplemented with 10% Fetal Bovine Serum (Capricorn, FBS-12B) and 1% Penicillin-Streptomycin was used [2]. To initiate differentiation, 2×10⁴ cells were seeded in each well of a 24-well plate. Differentiation of 3T3-L1 preadipocytes into mature adipocytes was carried out in 24-well plates. When the cells reached approximately 70% confluence, a pre-differentiation medium was applied. This medium consisted of 89% DMEM, 10 % Fetal Bovine Serum, and 1% Penicillin-Streptomycin, enriched with 1.0 µM dexamethasone (Sigma, BCBV5460), 0.5 mM isobutylmethylxanthine (IBMX; Sigma, STBF6061V), and 1.0 µg/mL insulin (Sigma, SLBV1793). The cells were incubated in this medium for 48 hours. After incubation, the pre-differentiation medium was replaced with a differentiation medium containing 1.0 µg/mL insulin. The differentiation medium was refreshed every 48 hours to maintain optimal nutrient levels. Throughout the differentiation process, morphological changes and lipid accumulation were monitored using microscopic observations. The incubation process continued until the 10th day, with the medium being replaced every alternate day. On the 12th day, the differentiation experiment concluded. Differentiation was considered complete when 90% of the cells exhibited the mature adipocyte phenotype, which was confirmed using Oil Red O staining.

**Microscopic analysis of changes in cell viability and morphology**

A precise seeding of 2x10^4^ cells was carried out, distributing them evenly across individual wells within a 24-well plate, and incubated for 24 hours in a humidified atmosphere with 5% CO_2_. Subsequently, the wells were enriched with a medium containing *U. dioica* extracts at concentrations of 50 μg/mL, 100 μg/mL, and 200 μg/mL. Following this setup, the cells underwent incubation for 24, 48, and 72 hours. After the specified incubation periods, a thorough examination of cell adhesion to the culture surface, as well as an assessment of their viability and morphological characteristics, was meticulously performed using an inverted microscope.

**LPS-induced inflammation in *U. dioica* treated adipocytes**

Differentiated 3T3-L1 adipocytes were exposed to lipopolysaccharide (LPS) at a concentration of 10 ng/mL (L6529, Escherichia coli O55:B5, Sigma) to establish an inflammatory model. Concurrently, varying concentrations of *U. dioica* extract (50, 100, and 200 μg/mL) were administered to evaluate its potential modulatory effects on LPS-induced inflammation. The LPS-containing medium was prepared by dissolving 1 mg of LPS in 1 mL of ultrapure water, followed by precise dilution to achieve a final concentration of 10 ng/mL in the culture medium. After a 60-minute incubation with LPS, *U. dioica* extract was introduced at the specified concentrations, with treatments applied at 24, 48, and 72-hour intervals. LPS, a well-characterized endotoxin composed of lipid A, core oligosaccharide, and O-antigen, triggers a potent inflammatory response via the activation of Toll-like receptor 4 (TLR4), which subsequently initiates a cascade of intracellular signaling events leading to the production of pro-inflammatory cytokines and other immune mediators. The selected LPS concentration of 10 ng/mL was carefully chosen based on established protocols and prior studies [3], balancing the induction of a robust inflammatory response with the avoidance of excessive cytotoxicity. Cellular samples were collected at 24, 48 and 72 hours post-application, facilitating subsequent analytical investigations into the cellular responses induced by LPS treatment.

**Visualizing lipid accumulation of 3T3-L1 adipocytes**

During 3T3-L1 adipocyte differentiation, we utilized the Biovision Oil Red O Staining Kit (Biovision, K580-24) to specifically stain neutral lipids, such as triglycerides. This lipophilic dye, known for its hydrophobic interaction with lipids, enabled the visualization of lipid droplets within cells. The staining procedure, involving fixation, permeabilization, and incubation with Oil Red O, followed the manufacturer's instructions. All treatments were performed using 500 μl in each well for a 24-well plate. First, the culture medium was removed from 3T3-L1 cells, and the cells were washed twice with PBS to remove any residual debris. Subsequently, the cells were fixed by incubating them with 10% formalin for 60 minutes. During the incubation period, a shaker was used to ensure homogenization. After fixation, the cells were incubated with 60% isopropanol for 5 minutes. Following the removal of isopropanol, the cells were incubated with Oil Red O solution (Biovision, Cat No: K580) for 15–20 minutes on a shaker. After incubation, the stain was removed, and the cells were washed 4–5 times with deionized water (dH₂O). Hematoxylin was then added to the cells and incubated for 1 minute, followed by its removal and another round of washing with dH₂O. The resulting lipid droplets appeared as red or orange-red structures under a microscope, providing insights into lipid accumulation during adipocyte differentiation. Following the treatment of adipocytes with LPS and varying concentrations of U. dioica extract, the Oil Red O staining protocol was utilized to evaluate cellular lipid accumulations. To quantitatively analyze lipid accumulation, the staining intensity was measured using image-specific software (ImageJ). Microscopic images of the quantitative analysis of Oil Red O staining are presented in Figure 2S.

**RNA extraction, cDNA Synthesis, and Real-Time PCR Analysis**

Total RNA was meticulously extracted from adipocyte cells using the advanced RiboExTM (Cat. No. 301-001) solution provided by GeneAll (Cat. No. 301-902). Rigorous assessments of the extracted RNA's quantity and purity were conducted through formaldehyde agarose gel electrophoresis and ultraviolet light spectrophotometry, respectively. In the subsequent step of complementary DNA (cDNA) synthesis, a refined approach was employed, with 500 nanograms of total RNA being expertly reverse transcribed. Using oligo-dT primers and the EasyScript Plus cDNA synthesis kit from ABM Alfagen, this procedure was carried out precisely (Cat. No. G236). The ensuing real-time PCR analysis was carried out using the renowned Roche Light Cycler 96 Real-Time PCR apparatus. The 45-cycle reaction mix contained Ampliqon RealQ Plus 2 Master Mix Green along with carefully crafted 0.3 M gene-specific forward and reverse primers. The thermal cycling parameters, involving denaturation, annealing, and extension, were set at 95°C, 55°C–58°C, and 72°C, respectively, each for a precise duration of 30 seconds. Maintaining the highest standards, the amplified 18S gene emerged as the reference standard for normalizing potential sample variations. Primer sequences for the targeted mouse genes, namely Dgat1, Lpl, Fas, Brca1, Brca2, Mcp1, and 18S RNA, were selected. The comprehensive information, including accession codes, for these primers was diligently curated from the National Center for Biotechnology Information (https://www.ncbi.nlm.nih.gov/genbank/). For a comprehensive overview of the primer sequences, please refer to Table S1. The comparative assessment between baseline and post-treatment measurements across groups was executed through the application of the t-test, with a predetermined significance level of 0.05. A t-test result falling below this threshold was deemed statistically significant in interpreting the outcomes.

**Molecular docking analysis**

The 2D chemical structures of ligands from the medicinal plant *U. dioica*, identified based on [1], were generated using ChemDraw 18.0 software. Optimizing geometric and electronic structures employed Gaussian 03 software with B3LYP/3-21G DFT and HF theory. Ligands were saved in Mol2 format and visualised using Chem3D 18.0. Then, using Autodock Tools 1.5.6, Mol2 files were converted to PDBQT format, integrating Gasteiger charges, combining nonpolar hydrogens, identifying aromatic carbons and rotatable bonds, and modifying TORSDOF values [4]. Protein structures corresponding to genes with statistically significant expression levels, as identified in the literature, were obtained in PDB format. The three-dimensional structures of key proteins, including Brca1 (PDB ID: 1T15) [5, 6,7], Brca2 (PDB ID: 1N0W)[8], Fas (PDB ID: 2PX6) [9], Mcp-1 (PDB ID: 1DOK) [10], Dgat1 (PDB ID: 6VP0) [11] and Lpl (PDB ID: 6E7K) [12] were retrieved from the Protein Data Bank (PDB; <https://www.rcsb.org/>). Autodock Tools 1.5.6 facilitated preprocessing, including visualization, removal of unwanted elements, and addition of hydrogens. Missing atoms were corrected, Kollman charges assigned, and imbalances distributed across residues [4,13].The refined structures were saved in PDBQT format for subsequent analysis [4]. Molecular docking studies of ligands on PDBQT-formatted protein structures were conducted using AutoDock Vina [14] via the DWIM Perl interface on a Windows 10 Home OS. AutoDock Vina employed Genetic Algorithm searches, generating twenty binding modes for each docking. The grid box defining the search space was adjusted with predefined coordinates (Table S2). Favorable positions were identified based on the lowest docking energies (kcal/mol). Selected conformations and structures were combined, analyzed using PyMOL and Discovery Studio Visualizer, and interaction patterns were presented in figures through screenshots. This streamlined approach enhances our understanding of molecular interactions for comprehensive scientific analysis.

**Abbreviations**  ALA: Alanine, ARG: Arginine, ASN: Asparagine, Bcl-2: B-cell lymphoma 2, BMI: Body mass index, BRCA1: Breast Cancer -1, BRCA2: Breast Cancer-2, CRP: C-reactive protein, CYS: Cysteine, Dgat1: Diacylglycerol-O-acyltransferase, DMEM: Dulbecco's Modified Eagles Medium, Fas: Fatty acid synthase, ERK: Extracellular Signal-Regulated Kinase, FBS: Fetal Bovine Serum; FCS: Fetal Calf Serum, FFA: Free fatty acids, JAK2/STAT3: Janus Kinase 2 / Signal Transducer and Activator of Transcription 3, GLN: Glutamine, GLU: Glutamic acid, HIS: Histidine, IBMX: Isobuthylmethilxsantin, IL-1β : Interleukin-1 beta, IL-6: Interleukin-6, ILE: Isoleucine, LEU: Leucine, Lpl: Lipoprotein lipase, LPS: Lipopolysaccharide, LYS: lysine, Mcp1: Monocyte Chemoattractant protein 1, mRNA: Messenger RNA, NADPH: Nicotinamide Adenine Dinucleotide Phosphate, NF-kB: Nuclear factor-KB, NIH: National Institutes of Health, PI3K/AKT: Phosphoinositide 3-Kinase / Protein Kinase B , PRO:Proline, qPCR:Quantitative PCR, SER: Serine, THR: threonine, TLR4: Toll-like Receptor 4, TNF-α: Tumor necrosis factor-α, TRP: tryptophan, TYR: tyrosine, *U. dioica*: *Urtica dioica* L., VAL: valine, WHO: World health organization

**References**

1. Repajić M, Cegledi E, Zorić Z et al (2021) Bioactive Compounds in Wild Nettle (Urtica dioica L.) Leaves and Stalks: Polyphenols and Pigments upon Seasonal and Habitat Variations. Foods 10:190. <https://doi.org/10.3390/foods10010190>

2. Pektaş M, Kurt AH, Ün I et al (2015) Effects of 17β-estradiol and progesterone on the production of adipokines in differentiating 3T3-L1 adipocytes: Role of Rho-kinase. Cytokine 72:130–134. https://doi.org/10.1016/j.cyto.2014.12.020

3. Cranmer-Byng MM, Liddle DM, De Boer AA et al (2015) Proinflammatory effects of arachidonic acid in a lipopolysaccharide-induced inflammatory microenvironment in 3T3-L1 adipocytes in vitro. Applied Physiology, Nutrition, and Metabolism 40:142–154. <https://doi.org/10.1139/apnm-2014-0022>

4. Kumar A, Bora U (2014) Molecular docking studies of curcumin natural derivatives with DNA topoisomerase I and II-DNA complexes. Interdiscip Sci Comput Life Sci 6:285–291. https://doi.org/10.1007/s12539-012-0048-6

5. Chakraborty A, Katarkar A, Chaudhuri K et al (2013) Detection of a novel mutation in exon 20 of the BRCA1 gene. Cell Mol Biol Lett 18:631–638. <https://doi.org/>10.2478/ s11658-013-0110-3

6. Muthusamy K, Kirubakaran P, Singh KD et al (2011) Molecular docking studies of Bitter melon compounds against BRCA1 protein. J. Pharm. Res 4:388–390

7. Prabhavathi H, Dasegowda KR, Renukananda KH et al (2021) Exploration and evaluation of bioactive phytocompounds against BRCA proteins by in silico approach. J Biomol Struct Dyn 39:5471-5485. https://doi.org/10.1080/07391102.2020.1790424

8. Khan MKA, Akhtar S, Arif JM (2018) Structural Insight into the Mechanism of Dibenzo [a,l] pyrene and Benzo [a] pyrene-Mediated Cell Proliferation Using Molecular Docking Simulations. Interdiscip Sci 10:653–673. https://doi.org/10.1007/s125390170226-7

9. Luna-Vital D, Weiss M, Gonzalez de Mejia E (2017) Anthocyanins from Purple Corn Ameliorated Tumor Necrosis Factor-α-Induced Inflammation and Insulin Resistance in 3T3-L1 Adipocytes via Activation of Insulin Signaling and Enhanced GLUT4 Translocation. Mol Nutr Food Res 61:1700362. https://doi.org/10.1002/mnfr.201700362

10. Piccinini AM, Knebl K, Rek A et al (2010) Rationally evolving MCP-1/CCL2 into a decoy protein with potent anti-inflammatory activity in vivo. Journal of Biological Chemistry 285:8782–8792. https://doi.org/10.1074/jbc.M109.043299

11. Yu J, Liao PJ, Xu W et al (2021) Structural model of PORCN illuminates disease-associated variants and drug binding sites. J Cell Sci*:* jcs259383. [https://doi.org/10.1242/ jcs.259383](https://doi.org/10.1242/jcs.259383)

12. Birrane G, Beigneux AP, Dwyer B et al (2019) Structure of the lipoprotein lipase–

GPIHBP1 complex that mediates plasma triglyceride hydrolysis. Proceedings of the

National Academy of Sciences 116:1723–1732.

<https://doi.org/10.1073/pnas.1817984116>

13. Morris GM, Goodsell DS, Halliday RS et al (1998) Automated docking using a Lamarckian genetic algorithm and an empirical binding free energy function. J Comput Chem 19:1639–1662. [https://doi.org/10.1002/(SICI)1096-987X(19981115)19:14< 1639 ::AID-JCC10>3.0.CO;2-B](https://doi.org/10.1002/(SICI)1096-987X(19981115)19:14%3c%201639%20::AID-JCC10%3e3.0.CO;2-B)

14. Trott O, Olson AJ (2010) AutoDock Vina: Improving the speed and accuracy of docking with a new scoring function, efficient optimization, and multithreading. J Comput Chem 31:455–61. <https://doi.org/10.1002/jcc.21334>

**List of tables**

**Table S1.** NCBI accession numbers and primer sequences for real-time PCR analysis

**Table S2.** Spatial parameters of the molecular docking grid box

**Table S3.** Administration of dosages of 50, 100, and 200 µg/ml *U. dioica* for 24 and 48 h and percentage changes in gene expression levels of Fas, Lpl, Dgat1, Mcp1, Brca1, and Brca2 genes compared with undifferentiated and differentiated controls in 3T3-L1 cells (U.d.) *p < 0.05, comparison with diffentiated control+LPS, +p < 0.05, comparison with nondifferentiation control.

**Table S4.** Protein-ligand interaction with the highest affinities

**Table S5.** Binding affinities and interaction profiles of the most effective ligands with BRCA1 (1T15), BRCA2 (1N0W), FAS (2PX6), MCP-1 (1DOK), DGAT-1 (6VP0) and LPL (6E7K).

**Table S1.** NCBI accession numbers and primer sequences for real-time PCR analysis

| Accesion Number | Gene | Primer sequence |
| --- | --- | --- |
| NM_010046.3 | Mus musculus  Dgat1 | (F): 5′-CCTCAGCCTTCTTCCATGAG-3′  (R): 5′-ACTGGGGCATCGTAGTTGAG-3′ |
| NM_008509.2 | Mus musculus  Lpl | (F): 5′-ACTCGCTCTCAGATGCCCTA-3′  (R): 5′-TTGTGTTGCTTGCCATTCTC-3′ |
| NM_007988.3 | Mus musculus  Fas | (F): 5′-CTGAGATCCCAGCACTTCTTGA-3′  (R): 5′-GCCTCCGAAGCCAAATGAG-3′ |
| NC_000077.7 | Mus musculus  Brca1 | (F):5’TCAGAAGAAGAGCGGATAGAGAC-3’  (R): 5’-ACACAAACTCCGCATCTGTTTTA-3’ |
| NC_000071.7 | Mus musculus  Brca2 | (F):5’-TCAGTTCCTGAGGATTTCACC-3’  (R):5’-ACAAAGGTACAGATGGGACTG-3’ |
| NM_011333.3 | Mus musculus  Mcp1 | (F): 5’-AGGTGTCCCAAAGAAGCTGT-3’  (R): 5’-AAGACCTTAGGGCAGATGCAG-3’ |
| NR_003278.3 | Mus musculus  18S ribosomal RNA | (F): 5′-TTCGAACGTCTGCCCTATCAA-3′  (R): 5′-ATGGTAGGCACGGCGACTA-3′ |

**Table S2.** Spatial parameters of the molecular docking grid box

| Proteins | PDB ID | Coordinates | Dimensions |
| --- | --- | --- | --- |
| Brca1 (Crystal Structure of BRCT Domain) | 1T15 | X= -7.73, Y= 24.32, Z= 38.68 | 30x30x30 Å |
| Brca2 (Crystal structure of the RAD51-BRCA2 BRC repeat complex) | 1N0W | X= 32.15, Y= 22.98, Z= 0.753 | 30x30x30 Å |
| Fas (Crystal structure of the thioesterase domain of human fatty acid synthase) | 2PX6 | X= -1.99, Y= -5.83, Z= 15.34 | 40x40x40 Å |
| Mcp1 (Monocyte Chemoattractant Protein 1, P-Form) | 1DOK | X= 21.84, Y= 39.12, Z= 34.91 | 30x30x30 Å |
| Dgat1 (Human Diacylglycerol Acyltransferase 1) | 6VP0 | X= 111.3, Y= 111.4, Z= 107.5 | 60x60x60 Å |
| Lpl (Structure of the lipoprotein lipase GPIHBP1 complex that mediates plasma triglyceride hydrolysis) | 6E7K | X= 16.53, Y= 16.41, Z= 3.496 | 60x60x60 Å |

**Table S3.** Administration of dosages of 50, 100, and 200 µg/ml *U. dioica* for 24 and 48 h and percentage changes in gene expression levels of Fas, Lpl, Dgat1, Mcp1, Brca1, and Brca2 genes compared with undifferentiated and differentiated controls in 3T3-L1 cells. (*U.d*.) *p < 0.05, comparison with diffentiated control+LPS, ^+^p < 0.05, comparison with nondifferentiation control.

| Samples genes of interest | Nondifferentiated control | Differentiated control +LPS | 50 µg/ml U.d+LPS | 100 µg/ml U.d+LPS | 200 µg/ml U.d +LPS |
| --- | --- | --- | --- | --- | --- |
| Fas 24 h | 73.8+19.5 | 100^+^ | 30.7*+5.7 | 134.4+42.4 | 281.4+233.1 |
| Lpl 48 h | 7.1+3.1 | 100^+^ | 12.8*+4.2 | 4.5*+1.4 | 47.0+21.4 |
| Dgat1 24 h | 28.1+8.2 | 100^+^ | 12.5*+5.2 | 29.7*+8.4 | 32.1+15.8 |
| Dgat1 48 h | 10.7+0.8 | 100^+^ | 20.2*+4.2 | 18.6* +2.4 | 193.4+72.9 |
| Mcp1 24 h | 16.9+7.6 | 100^+^ | 11.7*+4.2 | 42.9*+14.1 | 27.2*+12.2 |
| Mcp1 48 h | 16.0+4.4 | 100^+^ | 12.4*+2.7 | 9.1*+1.9 | 81.4+47.2 |
| Brca1 24 h | 49.5+12.8 | 100^+^ | 21.2*+6.2 | 22.2*+4.9 | 35.3*+4.8 |
| Brca2 24 h | 44.9+16.3 | 100^+^ | 18.9*+10.0 | 24.1*+7.8 | 22.2*+8.9 |

**Table S4.** Protein-ligand interaction with the highest affinities

| Proteins | BRCA1  (1T15) | BRCA2  (1N0W) | FAS  (2PX6) | MCP-1  (1DOK) | DGAT-1  (6VP0) | LPL  (6E7K) |
| --- | --- | --- | --- | --- | --- | --- |
| Ligands | **Molecular docking energy (kcal/mol)** | | | | | |
| Isorhamnetin rutinoside | -7.3 | -6.7 | -8.6 | -6.9 | **-10.3** | -8.2 |
| Quercetin acetyl rutinoside | -6.8 | -7.7 | **-10.3** | **-8.6** | -10.1 | -9.1 |
| Apigenin hexoside | **-7.5** | **-8.4** | -9.0 | -7.4 | -10.0 | **-9.2** |
| Kaempferol 3 O rutinoside | -7.0 | -6.6 | -9.4 | -7.6 | -9.9 | -8.7 |
| Quercetin 3 rutinoside | -6.9 | -6.5 | -8.1 | -6.7 | -9.7 | -8.8 |
| Kaempferol pentosyl hexoside | -7.1 | -6.9 | -8.8 | -7.0 | -9.6 | -8.3 |
| Procatechuic acid | -5.6 | -5.6 | -8.0 | -5.8 | -9.2 | -7.1 |
| Kaempferol hexoside | -7.0 | -7.7 | -8.8 | -7.0 | -9.0 | -8.0 |
| Quercetin 3 pentoside | -6.6 | -6.6 | -7.9 | -7.1 | -8.7 | -8.4 |
| Kaempferol 3 rhamnoside | -6.7 | -6.9 | -8.2 | -7.4 | -8.7 | -8.6 |
| Quercetin pentyl hexoside | -6.2 | -6.9 | -9.0 | -6.2 | -8.7 | -8.5 |
| Epigallocatechin gallate | -7.1 | -6.6 | -8.6 | -7.2 | -8.7 | -8.5 |
| Epicatechin gallate | -7.1 | -6.1 | -8.6 | -7.3 | -8.7 | -8.5 |
| Chlorogenic acid | -6.5 | -7.0 | -8.7 | -6.5 | -8.6 | -8.4 |
| Quercetin 3 rhamnoside | -6.6 | -7.0 | -8.3 | -6.6 | -8.6 | -8.6 |
| Quercetin acetyl hexoside | -6.4 | -6.2 | -8.5 | -6.8 | -8.6 | -8.6 |
| Apigenin | -6.4 | -6.5 | -8.5 | -7.6 | -8.6 | -8.4 |
| Naringenin | -6.3 | -6.8 | -8.5 | -7.6 | -8.6 | -8.3 |
| Luteolin | -6.6 | -6.7 | -8.8 | -7.7 | -8.4 | -8.2 |
| Quercetin | -6.5 | -6.5 | -8.3 | -7.5 | -8.3 | -8.2 |
| Kaempferol pentoside | -6.1 | -6.9 | -7.9 | -6.5 | -8.3 | -8.0 |
| Quercetin 3 glucoside | -6.3 | -6.2 | -7.7 | -6.5 | -8.2 | -8.1 |
| Isorhamnetin | -6.2 | -6.7 | -8.1 | -6.6 | -8.2 | -8.1 |
| Myricetin | -6.6 | -6.4 | -8.7 | -6.7 | -8.1 | -8.2 |
| Catechin | -6.9 | -6.5 | -9.0 | -6.9 | -8.1 | -7.6 |
| Kaempferol | -6.2 | -6.4 | -8.1 | -7.5 | -8.0 | -8.4 |
| Genistein | -6.1 | -6.3 | -7.9 | -7.0 | -8.0 | -8.9 |
| Epicatechin | -6.5 | -6.7 | -9.0 | -7.1 | -7.9 | -8.0 |
| Esculetin | -5.5 | -6.1 | -7.5 | -5.5 | -7.3 | -6.6 |
| Umbelliferone | -5.3 | -6.0 | -7.3 | -5.0 | -7.0 | -6.5 |
| Cinnamic acid | -5.4 | -5.2 | -7.5 | -4.7 | -6.6 | -5.9 |
| Scopoletin | -5.5 | -5.7 | -7.3 | -5.1 | -6.6 | -6.8 |
| Serotonin | -4.9 | -5.3 | -6.8 | -4.8 | -6.6 | -6.2 |
| P coumaric acid | -5.4 | -5.2 | -6.9 | -4.9 | -6.2 | -5.9 |
| Sinapic acid | -5.2 | -5.4 | -6.8 | -5.0 | -6.2 | -5.9 |
| Gentisic acid | -5.2 | -5.1 | -6.6 | -5.1 | -6.1 | -5.7 |
| Syringic acid | -4.8 | -5.3 | -6.5 | -4.8 | -6.1 | -5.7 |
| Gallic acid | -4.9 | -5.2 | -6.6 | -5.1 | -6.1 | -5.8 |
| Caffeic acid | -5.3 | -5.1 | -7.2 | -5.4 | -6.1 | -6.3 |
| Ferulic acid | -5.3 | -5.4 | -7.3 | -5.1 | -6.1 | -6.0 |
| P-hydroxybenzoic acid | -4.7 | -5.1 | -6.1 | -4.7 | -5.9 | -5.2 |
| Quinic acid | -4.7 | -5.3 | -6.9 | -5.0 | -5.8 | -5.9 |
| Acetycholine | -3.3 | -3.5 | -5.0 | -3.5 | -4.2 | -4.0 |
| Histamine | -3.2 | -4.0 | -4.8 | -3.5 | -4.1 | -4.2 |
| Formic acid | -2.5 | -2.7 | -2.8 | -2.4 | -2.8 | -3.0 |

**Table S5.** Binding affinities and interaction profiles of the most effective ligands with BRCA1 (1T15), BRCA2 (1N0W), FAS (2PX6), MCP1 (1DOK), DGAT1 (6VP0) and LPL (6E7K)

| **Ligands** | **Isorhamnetin rutinoside** | | **Quercetin acetyl rutinoside** | |
| --- | --- | --- | --- | --- |
| **Receptors** | **Binding Affinity (kcal/mol)** | **Interactions** | **Binding Affinity (kcal/mol)** | **Interactions** |
| BRCA1 (1T15) | -7.3 | Conventional Hydrogen Bond (GLY A:1656, ARG A:1699 and ASN A:1774)  Pi-Sigma (LEU A:1701)  Pi-Alkyl (LEU A:1679 and LYS A:1702) | -6.8 | Conventional Hydrogen Bond (GLY A:1656, ASN A:1678, LYS A:1702 and GLN A:1779)  Pi-Sigma (LEU A:1701)  Pi-Alkyl (LEU A:1701 and LYS A:1702) |
| BRCA2 (1N0W) | -6.7 | Conventional Hydrogen Bond (GLU A:308)  Alkyl (ARG A:167)  Pi-Alkyl (ALA A:331) | -7.7 | Conventional Hydrogen Bond (LEU A:112, GLN A:114, ALA A:295, THR A:298 and ASP A:316)  Carbon Hydrogen Bond (LEU A:112 and LEU A:113)  Amide-Pi Stacked (TYR A:315)  Alkyl (LEU A:113)  Pi-Alkyl (ARG A:299) |
| FAS  (2PX6) | -8.6 | Conventional Hydrogen Bond (ARG A:2413, SER A:2417, ARG A:2421, ALA B:2277 and TYR B:2425)  Carbon Hydrogen Bond (GLU A:2394)  Pi-Anion (GLU A:2394)  Pi-Pi Stacked (TYR B:2425)  Alkyl (VAL A:2397, ALA A:2398 and VAL A:2401)  Pi-Alkyl (ARG B:2275) | **-10.3** | Conventional Hydrogen Bond (ARG A:2275, GLU A:2395, SER A:2417, ARG A:2421, ALA B:2277, TRY B:2425 and ARG B:2428)  Carbon Hydrogen Bond (ASP B:2280)  Pi-Pi Stacked (TYR B:2425)  Alkyl (ARG A:2421)  Pi-Alkyl (PHE A:2418 and LEU B:2279) |
| MCP1 (1DOK) | -6.9 | Conventional Hydrogen Bond (ALA A:7 and ARG A:30)  Carbon Hydrogen Bond (THR A:32 and SER A:33)  Alkyl (ILE A:31)  Pi-Alkyl (VAL A:9 and LYS A:35) | **-8.6** | Conventional Hydrogen Bond (LYS B:38)  Carbon Hydrogen Bond (LYS A:35 and PRO A:37)  Pi-Pi T-Shaped (TYR A:13)  Pi-Alkyl (LYS B:35 and PRO B:37) |
| DGAT1 (6VP0) | **-10.3** | Conventional Hydrogen Bond (TRP C:377, ASN C:378, CYS C:385 and HIS C:415)  Carbon Hydrogen Bond (GLN C:375)  Pi-Pi Stacked (TRP C:374)  Pi-Pi T-Shaped (TRP C:377)  Alkyl (CYS C:385, ILE C:386, VAL C:407 and MET C: 434)  Pi-Alkyl (TRP C:374, TYR C:390 and PHE C:408) | -10.1 | Conventional Hydrogen Bond (ASN C:378 and SER C:411)  Carbon Hydrogen Bond (TRP C:374)  Pi-Pi Stacked (TRP C:374)  Pi-Pi T-Shaped (PHE C:408)  Pi-Alkyl (MET C:434) |
| LPL  (6E7K) | -8.2 | Conventional Hydrogen Bond (TYR B:121 and TYR B:158)  Carbon Hydrogen Bond (PRO B:187)  Pi-Pi Stacked (TRP B:82 and TYR B:121)  Alkyl (LEU B:160 and LYS B:265)  Pi-Alkyl (TRP B:82, TYR B:121, PRO B:187 and ILE B:221) | -9.1 | Conventional Hydrogen Bond (SER A:422, TRP B:82, SER B:159, ARG B:219, ILE B:221, ASP B:261 and LYS B:265)  Carbon Hydrogen Bond (PRO B:187 and HIS B:268)  Pi-Pi Stacked (TYR B:121)  Pi-Pi T-Shaped (TRP B:82)  Alkyl (ILE B:221)  Pi-Sigma (ILE B:221)  Pi-Alkyl (PRO B:187, ILE B:221 and LYS B:265) |

| **Ligands** |  | **Apigenin hexoside** |
| --- | --- | --- |
|  |  |  |
| **Receptors** | **Binding Affinity (kcal/mol)** | **Interactions** |
| BRCA1 (1T15) | **-7.5** | Conventional Hydrogen Bond (SER A:1655, LEU A:1657, ASN A:1678, THR A:1700 and LYS A:1702)  Pi-Cation (LYS A:1702)  Pi-Sigma (LEU A:1701)  Pi-Alkyl (LYS A:1702) |
| BRCA2 (1N0W) | **-8.4** | Conventional Hydrogen Bond (HIS A:294, SER A:296, THR A:297 and ARG A:299)  Pi-Donor Hydrogen Bond (SER A:317)  Pi-Alkyl (ILE A:314 and ALA A:323) |
| FAS  (2PX6) | -9.0 | Conventional Hydrogen Bond (ARG A:2275, ARG A:2421, ASP B:2280 and ARG B:2428)  Carbon Hydrogen Bond (ASP B:2280)  Pi-Cation (ARG A:2421 and ARG B:2428)  Pi-Anion (GLU A:2394)  Pi-Pi Stacked (TYR B:2425) |
| MCP1 (1DOK) | -7.4 | Conventional Hydrogen Bond (SER A:34, LYS A:38 and SER B:34)  Pi-Pi T-Shaped (TYR A:13)  Pi-Sigma (LYS B:35)  Pi-Alkyl ( PRO A:37and LYS B:35) |
| DGAT-1 (6VP0) | -10.0 | Conventional Hydrogen Bond (TRP C:374, ASN C:378, HIS C:382 and SER C:411)  Carbon Hydrogen Bond (TRP C:374)  Pi-Pi Stacked (TRP C:374)  Pi-Pi T-Shaped (PHE C:408)  Pi-Alkyl (MET C:434, ILE C:438 and ALA C:441) |
| LPL  (6E7K) | -9.2 | Conventional Hydrogen Bond (SER A:446, ARG A:447, GLU A:448, GLN A:454, ALA A:458 and GLU C:99)  Carbon Hydrogen Bond (HIS A:452)  Pi-Anion (GLU A:448)  Pi-Sigma (VAL A:461)  Pi-Alkyl (VAL A:461 and ARG A:447) |

**List of Figures**

**Fig. S1** Microscopic evaluation depicting cell adhesion, morphological features, and viability within 3T3-L1 cells following exposure to various concentrations (50 μg/mL, 100 μg/mL, and 200 μg/mL) of *U. dioica* extract, alongside a solvent control.

**Fig. S2** Evaluation of lipid accumulation in fully differentiated 3T3-L1 cells using Oil Red O staining.

**Fig. S3** Quantitative analysis was conducted to assess mRNA levels related to adipogenesis, focusing on key genes, namely Fas, Lpl, and Dgat1, Mcp1, Brca1 and Brca2 mRNA levels linked to breast cancer progression.

**Fig. S4** Exemplary docking outcomes depicting optimal interactions between target proteins and *U. dioica* extract ligands, highlighting key molecular binding events.


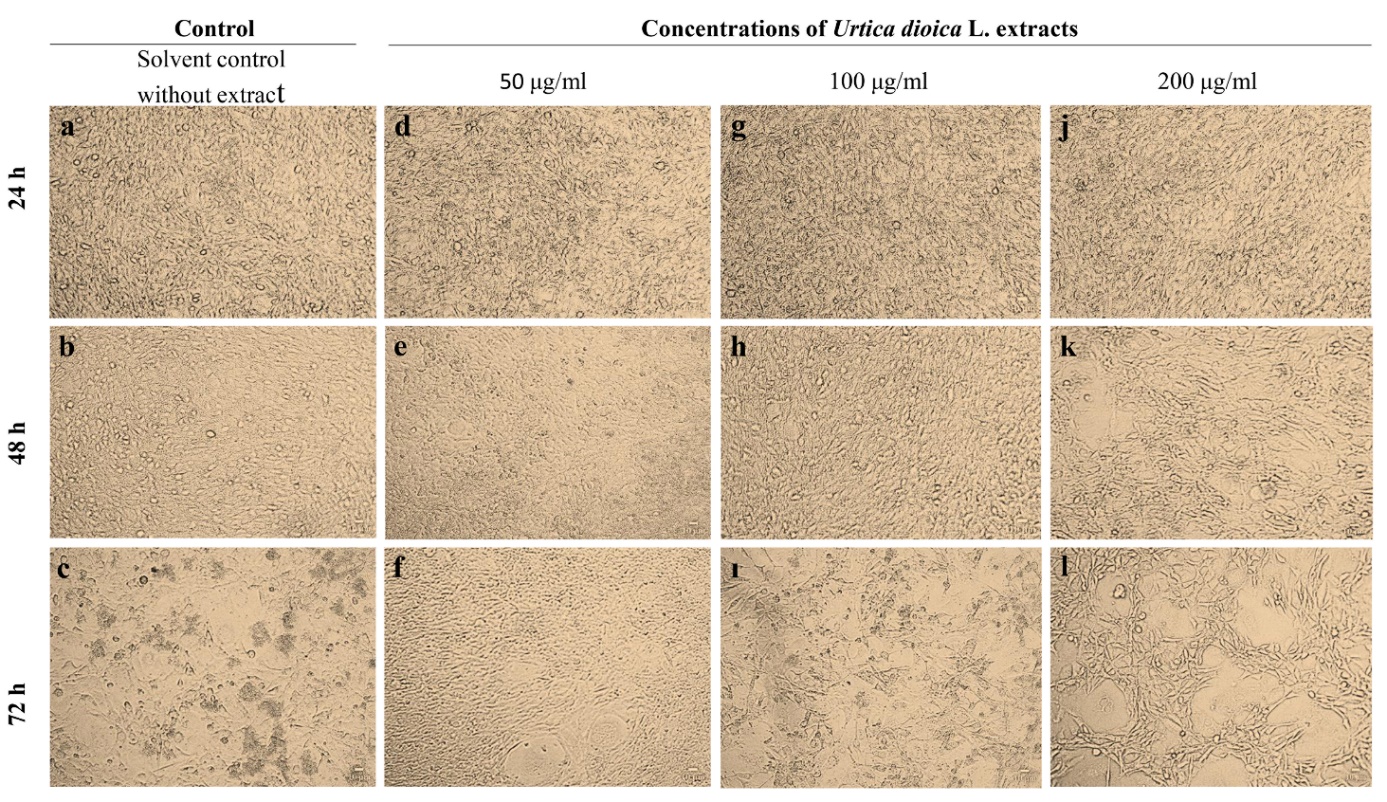


**Fig. S1** Microscopic evaluation depicting cell adhesion, morphological features, and viability within 3T3-L1 cells following exposure to various concentrations (50 μg/mL, 100 μg/mL, and 200 μg/mL) of *U. dioica* extract, alongside a solvent control. The assessment was performed at 24 h (a, d, g, j), 48 h (b, e, h, k), and 72 h (c, f, ı, l) post-treatment. Microscopic images captured at a 10x magnification.


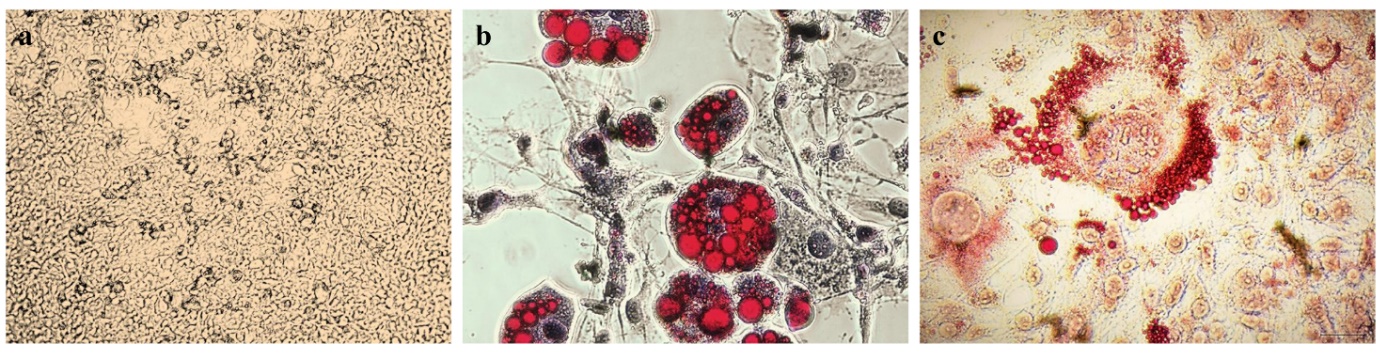


**Fig. S2** Evaluation of lipid accumulation in fully differentiated 3T3-L1 cells using Oil Red O staining. The images showcase cells before staining (a, 10x magnification) and post-staining (b, c, 40x magnification)


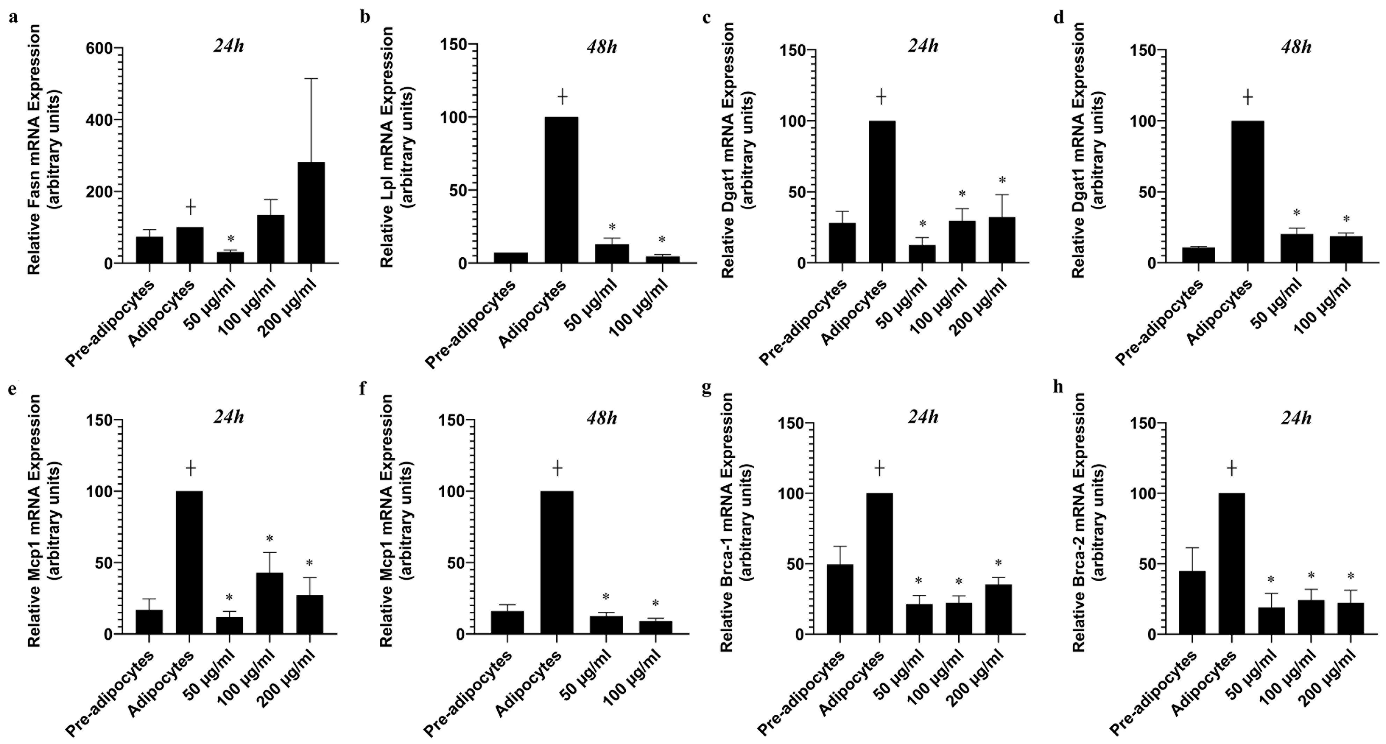


**Fig. S3** Quantitative analysis was conducted to assess mRNA levels related to adipogenesis, focusing on key genes, namely Fas, Lpl, and Dgat1 (a, b, c, d). Additionally, the study examined Mcp1 mRNA levels associated with inflammation (e, f), as well as Brca1 and Brca2 mRNA levels linked to breast cancer progression (g, h). *p < 0.05 compared to adipocytes (differentiation control). ┼ p < 0.05 compared to pre-adipocytes (non-differentiation control).


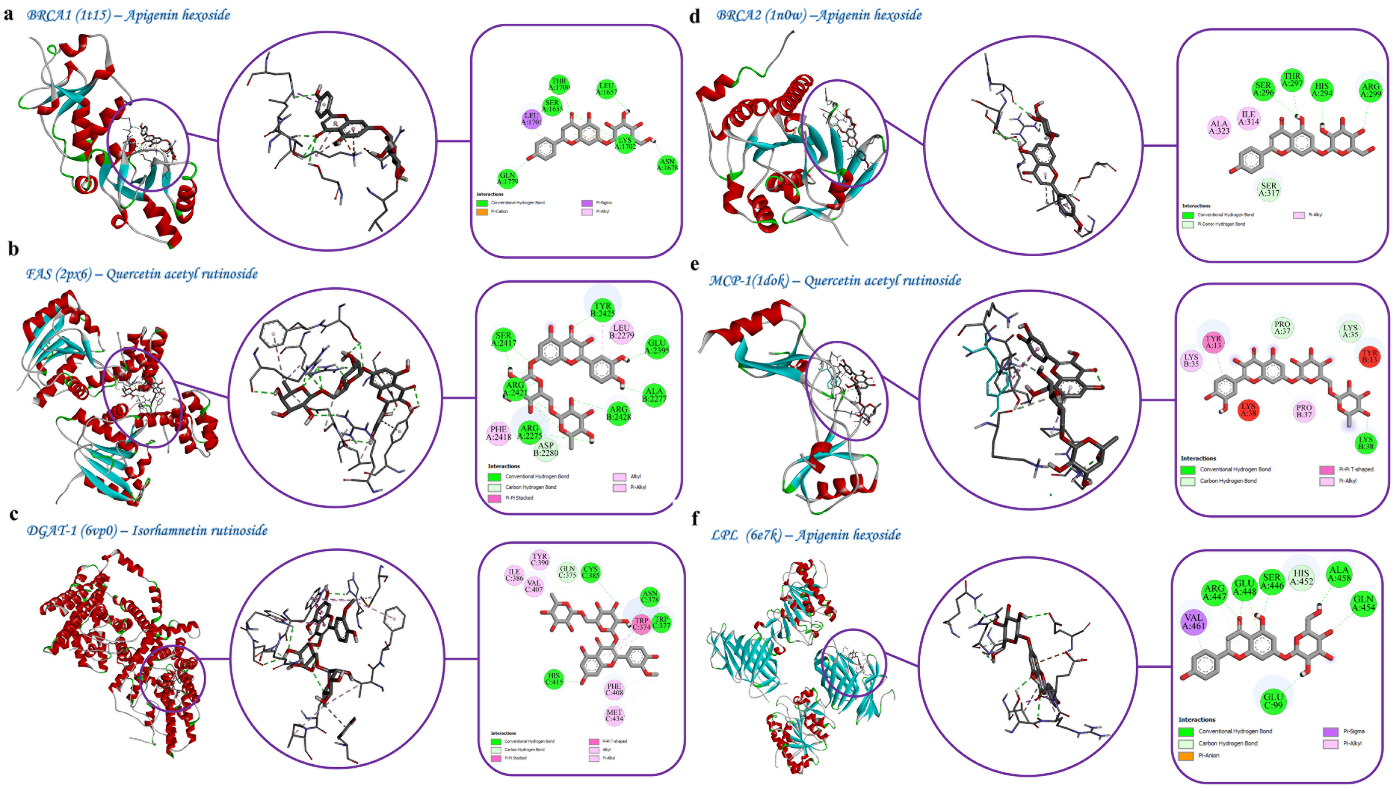


**Fig. S4** Exemplary docking outcomes depicting optimal interactions between target proteins Brca1(a), Brca2(d), Fas(b), Dgat1(c), Mcp1(e) and Lpl(f) and *U. dioica* extract ligands, highlighting key molecular binding events.
